# A Hematologist-Level Deep Learning Algorithm (BMSNet) for Assessing the Morphologies of Single Nuclear Balls in Bone Marrow Smears: Algorithm Development

**DOI:** 10.2196/15963

**Published:** 2020-04-08

**Authors:** Yi-Ying Wu, Tzu-Chuan Huang, Ren-Hua Ye, Wen-Hui Fang, Shiue-Wei Lai, Ping-Ying Chang, Wei-Nung Liu, Tai-Yu Kuo, Cho-Hao Lee, Wen-Chiuan Tsai, Chin Lin

**Affiliations:** 1 Division of Hematology/Oncology Department of Medicine Tri-Service General Hospital, National Defense Medical Center Taipei Taiwan; 2 Family Medicine Division Tri-Service General Hospital, National Defense Medical Center Taipei Taiwan; 3 Department of Pathology Tri-Service General Hospital, National Defense Medical Center Taipei Taiwan; 4 Graduate Institute of Life Sciences National Defense Medical Center Taipei Taiwan; 5 School of Public Health National Defense Medical Center Taipei Taiwan

**Keywords:** artificial intelligence, bone marrow examination, leukemia, myelodysplastic syndrome, deep learning

## Abstract

**Background:**

Bone marrow aspiration and biopsy remain the gold standard for the diagnosis of hematological diseases despite the development of flow cytometry (FCM) and molecular and gene analyses. However, the interpretation of the results is laborious and operator dependent. Furthermore, the obtained results exhibit inter- and intravariations among specialists. Therefore, it is important to develop a more objective and automated analysis system. Several deep learning models have been developed and applied in medical image analysis but not in the field of hematological histology, especially for bone marrow smear applications.

**Objective:**

The aim of this study was to develop a deep learning model (BMSNet) for assisting hematologists in the interpretation of bone marrow smears for faster diagnosis and disease monitoring.

**Methods:**

From January 1, 2016, to December 31, 2018, 122 bone marrow smears were photographed and divided into a development cohort (N=42), a validation cohort (N=70), and a competition cohort (N=10). The development cohort included 17,319 annotated cells from 291 high-resolution photos. In total, 20 photos were taken for each patient in the validation cohort and the competition cohort. This study included eight annotation categories: erythroid, blasts, myeloid, lymphoid, plasma cells, monocyte, megakaryocyte, and unable to identify. BMSNet is a convolutional neural network with the YOLO v3 architecture, which detects and classifies single cells in a single model. Six visiting staff members participated in a human-machine competition, and the results from the FCM were regarded as the ground truth.

**Results:**

In the development cohort, according to 6-fold cross-validation, the average precision of the bounding box prediction without consideration of the classification is 67.4%. After removing the bounding box prediction error, the precision and recall of BMSNet were similar to those of the hematologists in most categories. In detecting more than 5% of blasts in the validation cohort, the area under the curve (AUC) of BMSNet (0.948) was higher than the AUC of the hematologists (0.929) but lower than the AUC of the pathologists (0.985). In detecting more than 20% of blasts, the AUCs of the hematologists (0.981) and pathologists (0.980) were similar and were higher than the AUC of BMSNet (0.942). Further analysis showed that the performance difference could be attributed to the myelodysplastic syndrome cases. In the competition cohort, the mean value of the correlations between BMSNet and FCM was 0.960, and the mean values of the correlations between the visiting staff and FCM ranged between 0.952 and 0.990.

**Conclusions:**

Our deep learning model can assist hematologists in interpreting bone marrow smears by facilitating and accelerating the detection of hematopoietic cells. However, a detailed morphological interpretation still requires trained hematologists.

## Introduction

### Background

Bone marrow aspiration and biopsy have been the gold standard for diagnosing hematological diseases for decades. This procedure may be performed in the clinic for many conditions, such as anemia, leukopenia, leukocytosis, thrombocytopenia, thrombocytosis, pancytopenia, polycythemia, and hemochromatosis, as well as malignant diseases of the blood or bone marrow, which include leukemia, lymphoma, and multiple myeloma (MM), and fever of unknown origin [1]. Despite numerous new molecular markers and the development of new prognostic tools, bone marrow aspiration morphology remains a mandatory tool for disease diagnosis. A bone marrow specimen is collected and subsequently stained and interpreted by an experienced hematologist as a routine daily practice. The result interpretation is manpower consuming and operator dependent since years of training are required for a hematologist to become competent. It is a labor-intensive method for determining the differential count, and the obtained results show inter- and intravariations among specialists [2,3]. Therefore, it is important to develop a more objective and automated analysis system.

In addition to counting the cells in the bone marrow aspiration, the diagnosis and monitoring of leukemia disease severity via flow cytometry (FCM) [4] or molecular signatures [5] is becoming the standard of care and can guide our treatment plan setting. When a bone marrow specimen is obtained, the cells are stained with various CD markers for immunophenotyping to facilitate hematological diagnosis and prognostic prediction. Moreover, after induction chemotherapy, the bone marrow is typically aspirated again, and FCM is used to detect the leukemia-associated aberrant immunophenotype [6]. The current standard report for a bone marrow smear is based on manual counting and analysis of 300 or 500 cells, which is far fewer cells compared with FCM, which detects more than 100,000 events. However, detecting the immunophenotypes of the leukemia clone as minimal residual disease (MRD) via FCM is also complicated, and it is also dependent on the operator, antibody panel, protocol, and gating [7]. Furthermore, not all institutes have the facilities and the capability to monitor MRD accurately. We plan to overcome this weak point and establish a model of artificial intelligence (AI) assistance by recognizing many bone marrow smears to accumulate observed events and increase the accuracy and confidence in the detection of MRD by counting cells in the bone marrow smear.

With the AI revolution, several deep learning models have been developed for and applied to various areas of medical image analysis, such as chest X-ray interpretation [8], fundus photography [9], and skin lesion recognition [10]. These deep learning models can help physicians make diagnoses quickly and accurately. However, they have yet to be applied to hematological histopathology. Moreover, we are not satisfied with the direct use of deep learning models to classify the diagnoses of disease entities. Hematological histopathology differs from the histopathology of other diseases. Three main components are considered in hematological histopathology: the series of white blood cells (WBCs), erythrocytes, and megakaryocytes [11]. Commercial computer-aided diagnosis systems are available for peripheral blood sample recognition for clinical use [12]. However, no automated cell counting system is commercially available for bone marrow smears. Several difficult problems must be solved. First, blood cells in peripheral blood smears are much simpler to manipulate and easier to recognize as they contain only five types of WBCs: basophils, eosinophils, segmented neutrophils, monocytes, and lymphocytes. In contrast, bone marrow smears contain more cell types according to their stages of maturation. It will be difficult to identify each stage of the blood cells. Moreover, it is important to calculate the ratios of cell types for the diagnosis of hematological diseases. Second, the cell density in bone marrow smears is much higher than in peripheral blood smears; hence, the marrow sample is stickier. The cells are difficult to separate from one another, and many blood cells may cluster, which will hinder cell interpretation. Although the object detection deep learning model has rarely been used in medical research, its performance has been validated in other complex real-world scenarios [13]. We attempted to use this technology to overcome these two problems, and we believe that it could help us in daily clinical practice.

### Objectives

In this study, we retrieved previously evaluated bone marrow smear slides and the corresponding diagnoses, and we digitalized the films, which were divided into three cohorts: a development cohort, a validation cohort, and a competition cohort. We cropped and classified each cell from the development cohort and trained an object detection deep learning model. The cell-based performance of our deep learning model was compared with the performance of hematologists. Finally, patient-based validation was conducted to evaluate the correlation between AI predictions and clinical diagnosis by FCM.

## Methods

### Devolvement Cohort

The Tri-Service General Hospital, Taipei, Taiwan, provided the bone marrow smears from January 1, 2016, to December 31, 2016. Research ethics approval was granted by the Institutional Review Board for collecting data without individual consent (IRB No. 1-108-05-098). We selected 42 bone marrow smears from patients with a variety of diagnoses, which include leukemia, myelodysplastic syndrome (MDS), myeloproliferative disease (MPD), MM, aplastic anemia (AA), and lymphoma without bone marrow involvement, for the collection of lineages of cells (Table 1). We used a 1000× microscope and a camera to manually capture a total of 291 high-resolution photos for annotation (1920×2048). The annotation is based on a self-designed Web-based system, and the process includes (1) the constitution of cells by experienced technicians and (2) the classification of each cell into one of eight categories (erythroid, blasts, myeloid, lymphoid, plasma cells, monocyte, megakaryocyte, and unable to identify) by three independent hematologists. Finally, a total of 17,319 annotated cells were collected using the above process. Owing to the heterogeneity of classification among hematologists, the ground truth for cell classification is based on a majority decision. If three hematologists assign a single cell to inconsistent categories (1109/17,319; 6.40%), the ground truth is set as unable to identify. Moreover, we used a 6-fold cross-validation process to evaluate the model performance in object detection, in which each subsample cluster contains the images from 7 independent patients. No validation images belong to patients who have images that were used in the training. The final model that was used for further validation was trained on all 291 photos.

**Table 1:** Baseline characteristics in three study cohorts.

Baseline characteristics		Development cohort (N=42)		Validation cohort (N=70)		Competition cohort (N=10)	
**Gender, n (%)**							
	Female		22 (52)		37 (53)		4 (40)
	Male		20 (48)		33 (47)		6 (60)
Age (years), mean (SD)		57.8 (16.3)		56.5 (18.0)		46.9 (16.8)	
**Diagnosis, n (%)**							
	ALL^a^		7 (17)		7 (10)		2 (20)
	AML^b^		18 (43)		42 (60)		8 (80)
	MDS^c^		4 (10)		21 (30)		0 (0)
	AA^d^		2 (5)		0 (0)		0 (0)
	MM^e^		6 (14)		0 (0)		0 (0)
	MPD^f^		2 (5)		0 (0)		0 (0)
	Lymphoma		3 (7)		0 (0)		0 (0)

^a^ALL: acute lymphoblastic leukemia.

^b^AML: acute myeloid leukemia.

^c^MDS: myelodysplastic syndrome.

^d^AA: aplastic anemia.

^e^MM: myeloma.

^f^MPD: myeloproliferative disease.

### Validation Cohort

**Figure 1:**
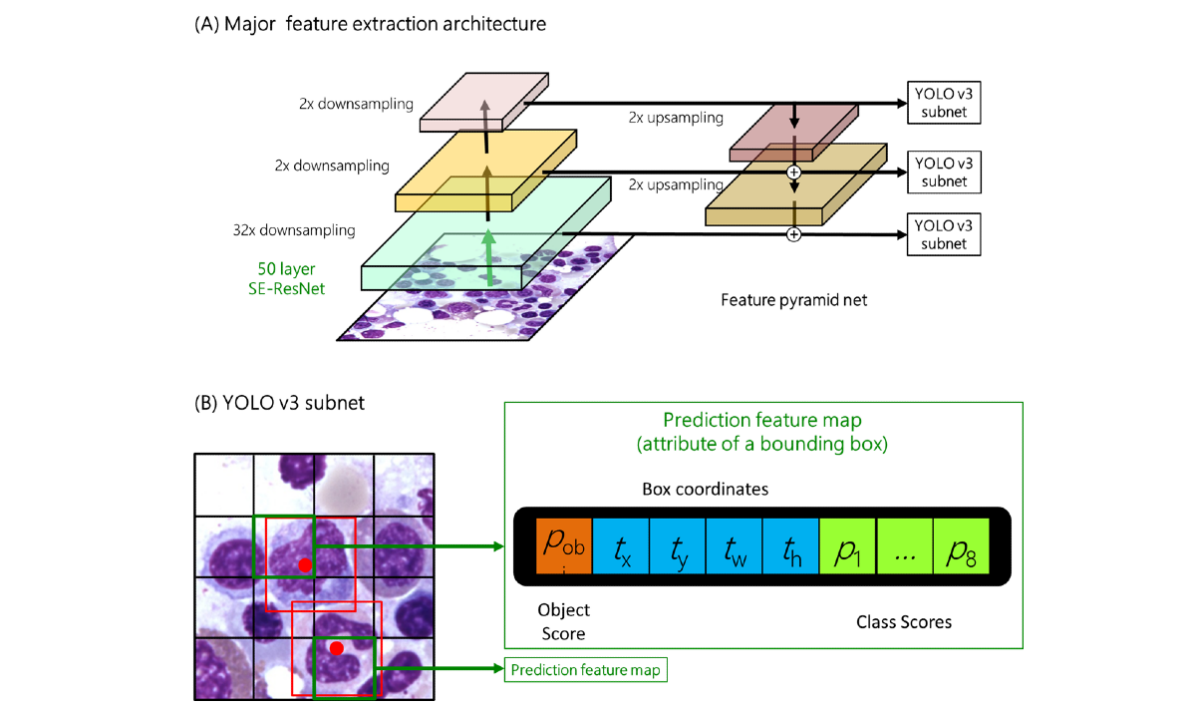
Deep learning model architecture. Our model contains a feature extraction architecture and a bounding box prediction subnet. The feature extraction architecture is based on a standard 50-layer SE-ResNet and a feature pyramid net, as illustrated in the upper half of the figure. The lower half illustrates the bounding box prediction process, which is based on the YOLO v3 architecture.

To validate the model performance in real-world clinical practice, we designed a validation cohort for evaluating the disease severity of leukemia and MDS. We included 70 bone marrow smears from January 1, 2017, to June 30, 2018, with acute leukemia and MDS before and after treatment. The model interpretation process is illustrated in Figure 1. Our technicians captured 20 photos for each case based on the above process, and these photos were analyzed using our model. The object detection model attempted to recognize all potential cells and to classify them. Finally, the model calculated the number and proportion of each kind of cell, except for the unable to identify category. We also collected the clinical interpretation reports from pathologists and hematologists in our hospital. According to the World Health Organization 2016 classification of myeloid malignancy, the diagnoses of MDS and acute leukemia mainly depend on the percentage of blasts [5]. The blast percentages of 5% to 9%, 10% to 19%, and more than 20% correspond to three disease statuses: MDS with excess blasts—1, MDS with excess blasts—2, and acute leukemia, respectively. Therefore, we classified the 70 cases into three categories (<5%, 5%-20%, and >20%) based on FCM.

### Competition Cohort

We further compared the performance between BMSNet and hematologists. A competition with 10 bone marrow smears was conducted with six visiting staff members. The independent bone marrow smears were from July 1, 2018, to December 31, 2018. The model interpretation process was similar to that for the validation cohort. The participants reviewed these 10 bone marrow smears under high magnification (1000×) to morphologically assess each cell.

### Bone Marrow Aspiration

After obtaining clinical informed consent, the patient was laid in the lateral decubitus position. The posterior superior iliac spine was prepped and draped in a sterile fashion. The crest of the posterior superior iliac spine was located, and the skin, along with the surface of the bone, was anesthetized with 2% lidocaine. A Kelly needle was introduced, and bone marrow aspirate was obtained.

### Bone Marrow Smear Staining and Digitalization

Bone marrow aspirate was evenly smeared across a sterile slide by a second slide and stained to air dry quickly. Next, 1.0 mL of the Wright-Giemsa stain was placed on the smear for 3 to 4 min. Then, 2.0 mL of distilled water or phosphate buffer of pH 6.5 was added, and it was left to stand for 6 to 8 min. The stained smear was rinsed with water until the edges showed a faint pinkish-red coloration. The film was allowed to dry in the air. All immunohistochemical stains were applied in the hematology laboratory of Tri-Service General Hospital. The images of the prepared slides were acquired at 1000× magnification with a BX53 light microscope (Olympus).

### Flow Cytometry

The RBCs were removed from the samples via fluorescence-activated cell sorting (FACS) lysis buffer. The cells were washed with FACS buffer, and a minimum concentration of 5×10^6^ cells/mL was obtained. The pellet from the final wash was resuspended and stained with various markers (Table 2: Panel). The panels were, then, sent for FACS analysis.

**Table 2:** Monoclonal antibodies: flow marker panels.

Tube	Fluorochromes			
	FITC^a^	PE^b^	PreCP^c^	APC^d^
1	Isotype	Isotype	CD45	N/A^e^
2	HLA-DR^f^	CD11b	CD45	N/A
3	CD19	CD5	CD45	N/A
4	CD56	CD38	CD45	N/A
5	CD16	CD13	CD45	N/A
6	CD15	CD34	CD45	N/A
7	CD14	CD33	CD45	N/A
8	CD7	CD56	CD45	N/A
9	HLA-DR	CD34	CD45	N/A
10	CD2	CD117	CD45	N/A
11	CD34	CD38	CD45	N/A
12	CD20	CD10	CD19	CD45
13	CD22	CD34	CD19	CD45
14	CD33	CD13	CD19	CD45
15	CD7	CD3	CD45	N/A
16	Isotype	Isotype	Cyto CD45	N/A
17	Cyto MPO^g^	Cyto TdT^h^	Cyto CD45	N/A

^a^FITC: fluorescein isothiocyanate.

^b^PE: phycoerythrin.

^c^PreCP: peridinin-chlorophyll.

^d^APC: allophycocyanin.

^e^Not applicable.

^f^HLA-DR: human leukocyte antigen–DR isotype

^g^MPO: myeloperoxidase.

^h^TdT: terminal deoxynucleotidyl transferase.

### Model Architecture

Suppose the input image is a 1000× photo with 1920×2048 pixels. To detect the potential cells, we used the YOLO v3 architecture to encode bounding boxes and construct the loss function [13]. Our deep learning model architecture is summarized in Figure 1. The major feature on which the extraction architecture is based is a 50-layer SE-ResNeXt [14], which won the ImageNet Large-Scale Visual Recognition Challenge in 2017. This SE-ResNeXt is pretrained by ImageNet, and the last feature map is saved for further use. The output features of SE-ResNeXt are downsampled by a factor of 32 compared with the original images. For example, the output feature shape is 60×64 when the shape of our input image is 1920×2048. Then, this feature map is passed through a convolutional module for further downsampling. The convolutional module consists of the following layers: (1) a 1×1 convolution layer (stride=1×1) with 1024 filters for reducing the dimensionality of the data, (2) a batch normalization layer for normalizing the input data, (3) a rectified linear unit (ReLU) layer for nonlinearization, (4) a 3×3 convolution layer (stride=2×1) with 1024 filters that belong to 64 groups for extracting features, (5) a batch normalization layer for normalization, (6) a ReLU layer for nonlinearization, (7) a 1×1 convolution layer (stride=1×1) with 2048 filters for recovering the dimensions, (8) a batch normalization layer for normalization, and (9) a ReLU layer for nonlinearization to extract features. The feature shapes are 30×32 and 15×16 after the first and second convolutional modules, respectively. Then, the three feature maps were passed through a feature pyramid net. The last feature was used directly for a YOLO v3 subnet and was passed through a deconvolutional module for upsampling at the same time. We constructed the deconvolutional module from the following layers: (1) a 2×2 deconvolution layer (stride=2×2) with 2048 filters for increasing the dimensions of the data, (2) a batch normalization layer for normalizing the input data, and (3) a ReLU layer for nonlinearization. After the deconvolutional operation, the shape of the second feature map and that of this upsampling feature map were similar; therefore, they were passed through an additional layer that was based on residual learning to integrate their information. This integrated feature was used for another YOLO v3 subnet. The largest feature map was generated by following the same approach: the previous feature map was passed through a deconvolutional module and an additional layer. Finally, three YOLO v3 subnets were predicting blood cells of different sizes separately.

The YOLO v3 subnet is a grid-wise prediction architecture for detecting a potential object. For each bounding box, we must find the corresponding grid that contains its center. Given that there are almost no overlapping cells in our task, we modified the original YOLO v3 architecture such that only one bounding box is predicted by each grid. The YOLO v3 subnet is based on each feature map and includes a 1×1 convolution layer with 13 filters for predicting the object score, box coordinates, and class scores. The object score (*p*_obj_) is defined as the probability that the grid contains the object center, which ranges from 0 to 1. If the center of an object falls into a grid, that grid is responsible for detecting that object. The box coordinates include four types of information that describe the bounding box. *t*_x_ and *t*_y_ are defined as relative coordinates inside each grid and range from 0 to 1. For example, the coordinates 0 and 0 correspond to the point in the top left, and the coordinates 0.5 and 0.5 correspond to the point in the center. *t*_w_ and *t*_h_ are defined as the offsets in the log scale between the bounding box and the *anchor box*. The *anchor box* is generated via clustering analysis that is based on YOLO v2 [15], and the small, middle, and large anchor boxes in our experiments are 136 (width), 143 (height) pixel, (183, 185), and (293, 242). Here, we define the width and height of the original bounding box as *b*_w_ and *b*_h_ respectively, and the width and height of the anchor boxes as *a*_w_ and *a*_h_, respectively. The relationships among *t*_w_, *t*_h_, *a*_w_, *a*_h_, *b*_w_, and *b*_h_ are expressed in the following equations: *b_w_=a_w_e^tw^* and *b_h_=a_h_e^th^*. Finally, there are eight class scores (*p*_1_ to *p*_8_) in our YOLO v3 subnet, which correspond to the eight categories of cells and whose values range from 0 to 1. The parameters that range from 0 to 1 were transformed by a sigmoid function, and the remaining parameters were simple linear outputs.

### Training Details

We used a software package, namely, MXNet version 1.3.0 [16], to implement our deep learning model in the R language. Here, we have prepared a tutorial in GitHub using an open database to enable the readers to easily repeat our work [17]. The settings that were used for the training model are as follows: (1) the stochastic gradient descent optimizer with 0.005 learning rate and 0.9 momentum for optimization, (2) a bench size of 2, and (3) a weight decay of 10^−4^ [18]. Moreover, a few augmentation methods were used in our training process owing to the many parameters in the deep learning architecture relative to the sample size: (1) horizontal and vertical flipping at random, (2) random cropping of original images to a size of 1408×1536, and (3) random color transformation. All detailed settings can be found in our GitHub repository. We had explored a series of thresholds to optimize the model performance; however, the results demonstrated the robustness of the threshold selection in our task. Therefore, the threshold of the probability score of bounding box objects was set as 0.5 based on convention.

### Statistical Analysis and Model Performance Assessment

We presented the model characteristics as the means and standard deviations, numbers of patients, or percentages, as appropriate. We used a significance level of *P*<.05 throughout the analysis. The statistical analysis was carried out using the software environment R version 3.4.3.

In the development cohort, the first analysis was an evaluation of the accuracies of the hematologists in terms of precision and recall. In medical terminology, precision and recall refer to the positive predictive value and the sensitivity, respectively. The second analysis was an evaluation of the consistency between the hematologists and AI in terms of Cohen kappa coefficient. The third analysis was an evaluation of the deep learning model performance in terms of the average precision. A successful prediction must have more than 50% intersection over union (IoU) compared with the ground truth. The average precision based on the area under the curve (AUC) of the precision-recall curve is the most commonly used index for evaluating object detection models; therefore, we presented the average precision values for each cell category. However, the objective of an object detection model is to recognize the class correctly and to present the bounding boxes; therefore, we also presented the precision and recall after excluding the bounding box error. The bounding box error does not affect the clinical utility because we only focus on the proportions among the cells in practice. All model performance indicators were calculated based on 6-fold cross-validation.

**Figure 2:**
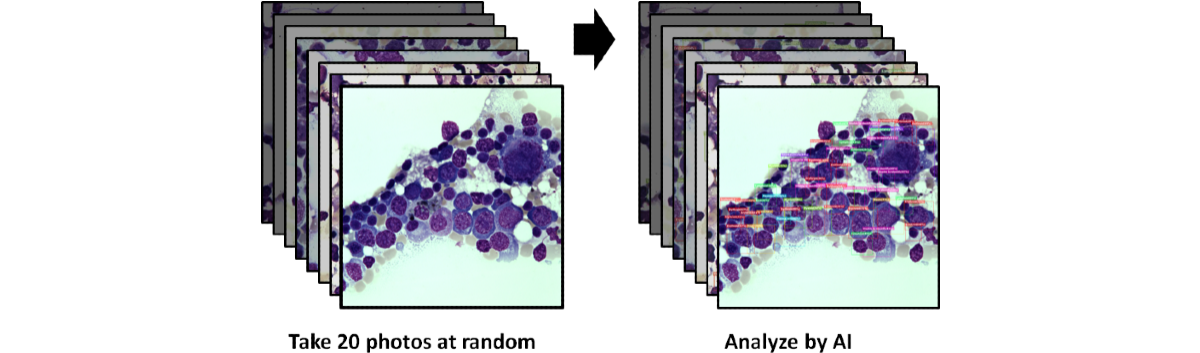
Artificial intelligence interpretation process. This flow chart demonstrates how to use BMSNet in clinical practice. In all, 20 photographs of each bone marrow smear slide are taken at random, and BMSNet will provide a cell-based prediction for each image. Finally, the total proportion of each category of cells is calculated based on cell counts.

The analysis for evaluating the AI model performance in clinical practice comprises three parts. First, we used the receiver operating characteristic (ROC) curve to evaluate the treatment efficacy evaluation accuracy for acute leukemia in the validation cohort. As patients with more than 5% vs 20% blast proportions required different treatment strategies, we presented the ROC curves that are based on these two cut points simultaneously to compare the performances of BMSNet, pathologists, and hematologists. Second, a competition on 10 smears was used to compare the consistency between the deep learning model and each hematologist. The output format, which is demonstrated in Figure 2, is a list of the proportions of seven categories (excluding unable to identify) in each bone marrow smear; therefore, we compared the correlation coefficients between the proportions that were obtained by the hematologists and deep learning model in each case. Third, the FCM was used to validate the proportions of four categories: blasts, myeloid, lymphoid, and monocyte. The correlation coefficients between these four proportions according to FCM results and the proportions that were obtained by the algorithm or hematologists were also presented as the mean values with 95% CIs. The paired *t* test was used to test these 10 correlations between the physicians and the AI model.

## Results

### Development Stage

The baseline characteristics of the development cohort, the validation cohort, and the competition cohort are presented in Table 1. The development cohort comprised 22 women and 20 men with a mean age of 57.8 (SD 16.3) years. The proportions of acute lymphoblastic leukemia (ALL), acute myeloid leukemia (AML), MDS, AA, MM, MPD, and lymphoma were 17% (7/42), 43% (18/42), 10% (4/42), 5% (2/42), 14% (6/42), 5% (2/42), and 7% (3/42), respectively. The validation cohort contained only ALL (7/70, 10%), AML (42/70, 60%), and MDS (21/70, 30%) cases and comprised 37 women and 33 men with a mean age of 56.5 (SD 18.0) years. To evaluate the sensitivity in monitoring the treatment efficacy (for MRD), the competition cohort included only 20% (2/10) ALL cases and 80% (8/10) AML cases without any MDS cases. The additional diseases in the development cohort were because of rare lymphocytes, plasma cells, monocytes, and megakaryocytes in acute leukemia and MDS. However, we focused only on acute leukemia and MDS in the validation cohort and in the competition cohort. These two diseases are the most crucial for physicians to diagnose and to design treatment strategy for immediately.

**Figure 3:**
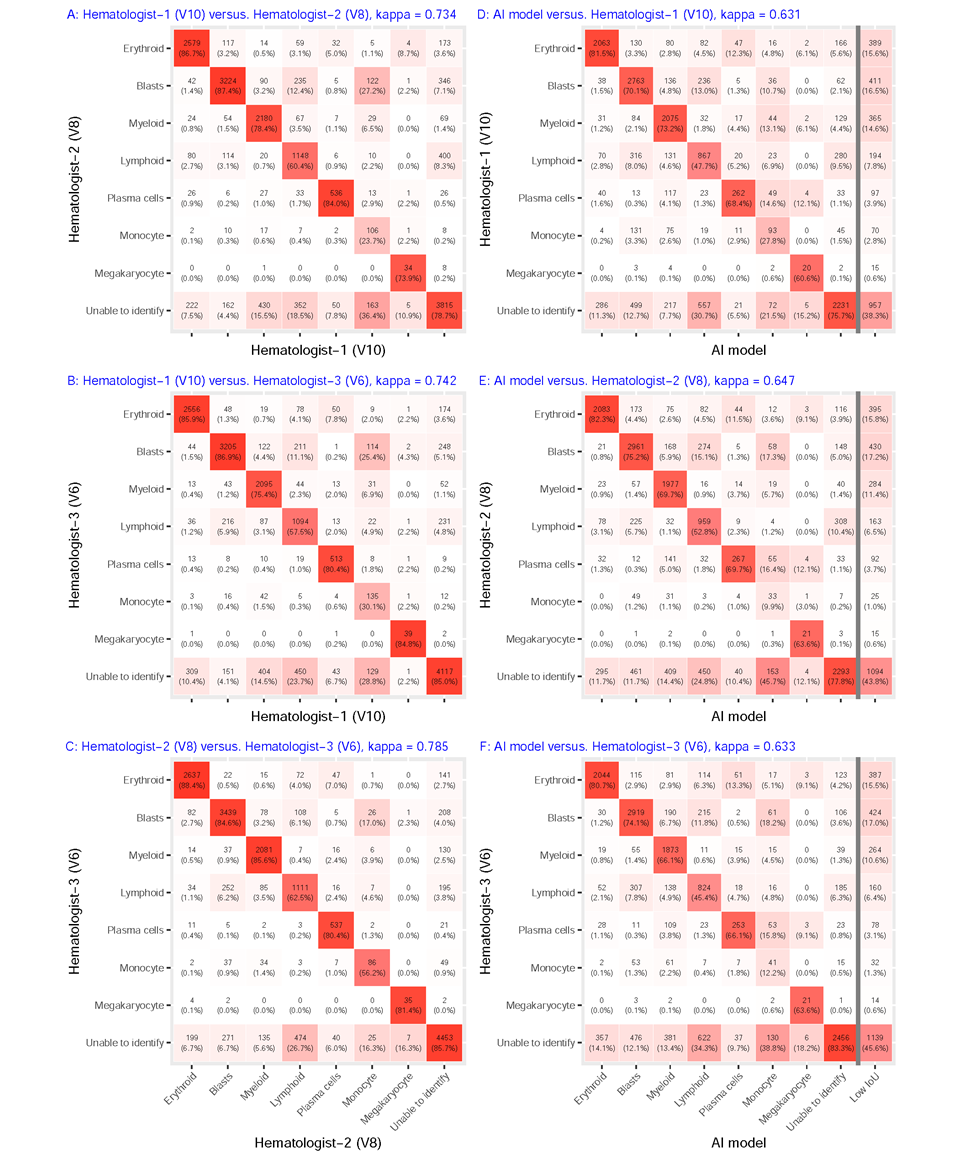
Cell-based consistency analysis in the development cohort. Each confusion matrix compares one of the three hematologists and AI. The kappa value is based on the eight-category classification, and 14.40% (2498/17,347) of cells that had lower IoUs were ignored in the AI-hematologist comparison. AI: artificial intelligence; IoU: intersection over union.

The cell classification performances are compared between the hematologists and BMSNet in Table 3. There were 17,319 cells in all 291 photos, and the numbers of cells that were classified as erythroid, blasts, myeloid, lymphoid, plasma cells, monocyte, megakaryocyte, and unable to identify are 2967, 4063, 2506, 1619, 600, 192, 42, and 5330, respectively. First, we evaluated the consistency among the three hematologists, and we found variations in the distributions of precision and recall among categories of cells. For example, monocyte recognition was the most difficult task for the three hematologists, with precision and recall results that ranged from 25.9% to 65.7% and 37.5% to 88.7%, respectively. In contrast, erythroid recognition was the easiest task, with precision and recall results that ranged from 87.6% to 89.1% and 92.3% to 91.4%, respectively. This demonstrated the difficulty of monocyte classification compared with erythroid classification. Fortunately, the intraclass average performances were similar, except for the megakaryocyte class. However, the distributions of precision and recall for the monocyte class differed among the hematologists. The precision and recall of hematologist-1 for the monocyte class were 25.9% and 88.7%, respectively, whereas the precision and recall of hematologist-2 were 65.7% and 37.5%, respectively. Hence, hematologist-1 was more likely to classify a cell as a monocyte, and hematologist-2 was more likely to make conservative identifications. Figure 3 presents the consistency analysis results among the three hematologists. The kappa values were 0.734 (V10 vs V8), 0.742 (V10 vs V6), and 0.785 (V8 vs V6). Strong inconsistencies in monocyte classification were observed compared with other categories, and the major misclassifications were because of an inability to distinguish among the blast, unable to identify, and monocyte classes.

A correct prediction by BMSNet consists of not only a correct classification but also a bounding box with more than 50% IoU. First, we evaluated the bounding box prediction performance, and the average precision without considering the classification was 67.4%. Hence, BMSNet might miss cells, which will lead to lower average precision in each category compared with the hematologists. However, the precisions and recalls of BMSNet were similar to those of the hematologists after we excluded the bounding box prediction error in most categories. BMSNet only performed at a large disadvantage considering the plasma cells, monocyte, and megakaryocyte classes compared with the hematologists. As each hematologist contributed one-third to the ground truth, the lower precisions and recalls that were realized by BMSNet are acceptable. Figure 3 presents the results of the consistency analysis between hematologists and BMSNet. The kappa values were 0.631 (V10), 0.647 (V8), and 0.633 (V6) when we ignored the cells with low IoU. The lymphoid and monocyte classes suffered from major misclassifications. The cells with low IoU were often classified as unable to identify by hematologists. On the basis of this observation, the proportions among the cells might be correct if we ignore the cells with low IoU or those of unable to identify.

**Figure 4:**
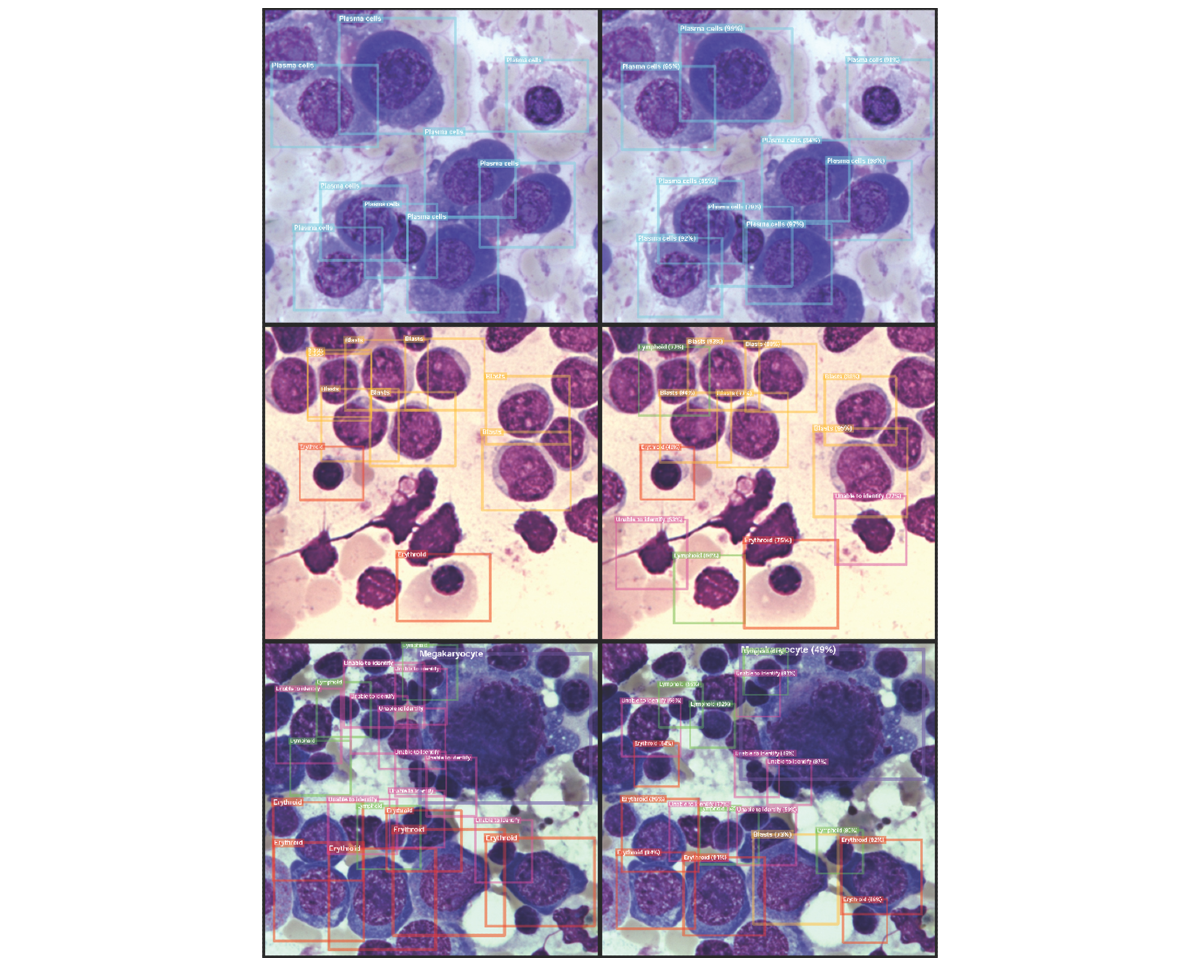
Multicell detection by the artificial intelligence model in selected views of bone marrow smear slides. The images in the left column are the consensus results from the hematologists in the morphological assessment of each cell, and the images in the right column are the predictions of BMSNet. From top to bottom are a myeloma case, an acute leukemia case, and a normal case. The colors of the bounding boxes correspond to the categories of the contained cells.

**Figure 5:**
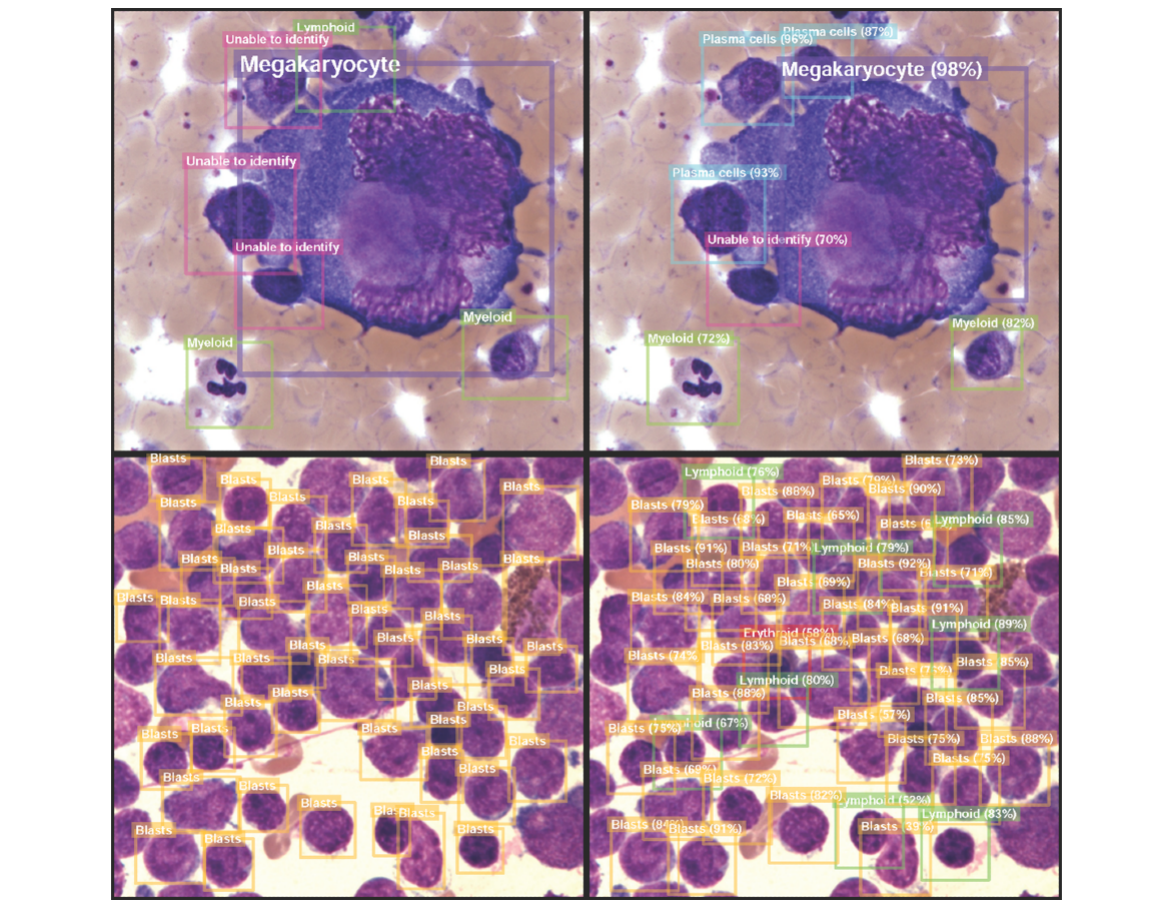
Selected inconsistent results between the hematologists and the artificial intelligence model. The images in the left column are the consensus from hematologists in the morphological assessment of each cell, and the images in the right column are the predictions of BMSNet. From top to bottom are a normal case and an acute leukemia case. The colors of the bounding boxes represent the categories of the contained cells.

Figure 4 shows selected views of consensus from the hematologists’ and BMSNet’s predictions. Most of the cells were correctly recognized by BMSNet; however, the predicted bounding boxes often did not match the ground truth perfectly. Moreover, cell debris was also recognized as cells; however, fortunately, the cell debris was often classified as unable to identify. As only the accurate proportion of each category of cells is needed in clinical practice, the bounding box prediction error might not affect the potential application of BMSNet in clinical practice. Figure 5 presents selected inconsistent results between the hematologists and BMSNet. When cells were close to each other, sometimes hematologists failed to identify them as a plasma cell, while BMSNet made the correct choice. Moreover, packed lymphoblasts were not easy to differentiate from lymphocytes. A case-based validation is conducted to evaluate the value of BMSNet in simulated clinical practice.

**Table 3:** Cell classification performances of hematologists and the deep learning model in the development cohort.

Cell class	Precision/recall/AP^a^ (%)			
	Hematologist-1 (V10)^b^	Hematologist-2 (V8)^b^	Hematologist-3 (V6)^b^	Artificial intelligence model^c^
Cells^d^ (n=17,319)	N/A^e^	N/A^e^	N/A^e^	55.8/85.6/67.4
Erythroid (n=2967)	87.6/92.3/N/A^e^	88.0/94.1/N/A	89.1/91.4/N/A	85.0/84.5/49.1
Blasts (n=4063)	91.0/85.2/N/A	79.1/88.2/N/A	87.5/88.5/N/A	86.5/80.7/50.2
Myeloid (n=2506)	79.1/94.2/N/A	92.0/93.5/N/A	93.8/80.0/N/A	94.0/76.4/49.5
Lymphoid (n=1619)	59.0/78.4/N/A	67.1/79.7/N/A	61.2/71.9/N/A	74.0/58.9/21.9
Plasma cells (n=600)	84.0/92.6/N/A	82.3/96.7/N/A	84.9/81.4/N/A	53.4/74.1/30.0
Monocyte (n=192)	25.9/88.7/N/A	65.7/37.5/N/A	40.2/64.5/NA	57.4/30.0/6.1
Megakaryocyte (n=42)	84.1/97.0/N/A	52.9/61.5/N/A	96.8/100.0/N/A	71.0/56.4/19.0
Unable to identify (n=5330)	86.5/78.5/N/A	82.3/77.5/N/A	83.9/93.5/N/A	60.9/86.1/25.1

^a^AP: average precision based on the area under the precision-recall curve.

^b^The abbreviation V(X) denotes a visiting staff member with (X) years of practice experience.

^c^All results were based on 6-fold cross-validation.

^d^Bounding box prediction performance regardless of the classifications (only for the deep learning model).

^e^Not available.

### Clinical Validation

**Figure 6:**
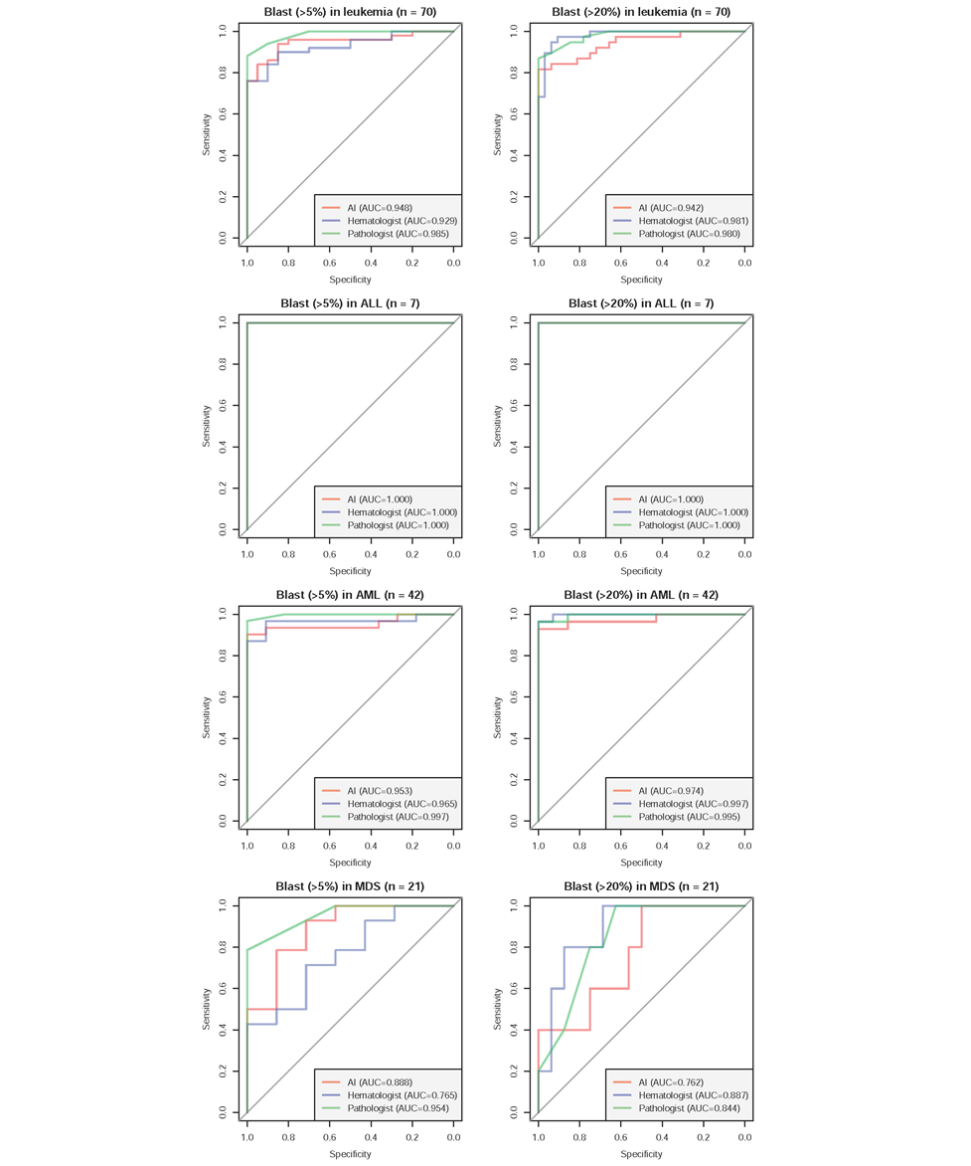
Receiver operating characteristic (ROC) curves in the diagnosis of acute leukemia and myelodysplastic syndrome in the validation cohort (n=70). The ROC curves correspond to the blast proportions that were obtained by BMSNet, the hematologists, and the pathologists. The outcome value is defined as more than 5%/20% blasts via flow cytometry. AI: artificial intelligence; ALL: acute lymphoblastic leukemia; AML: acute myeloid leukemia; AUC: area under the curve; MDS: myelodysplastic syndrome; ROC: receiver operating characteristic.

Figure 6 presents the ROC curves for the diagnosis of acute leukemia and MDS in the validation cohort. The analyses were conducted to two scenarios: the cutoff level blast percentage is 20% for diagnosing acute leukemia and 5% for treatment response monitoring. In detecting more than 5% of blasts, the AUC of BMSNet (0.948) was higher than that of the hematologists (0.929) in all leukemia cases, but lower than the AUC of the pathologists (0.985). In a further stratified analysis, we found that the source of the performance difference is MDS cases. The AUCs of BMSNet, the hematologists, and the pathologists in MDS cases were 0.888, 0.765, and 0.954, respectively. The performances of BMSNet, the hematologists, and the pathologists were similar in ALL and AML cases. Perfect AUCs of 100% for ALL cases were realized by BMSNet, the hematologists, and the pathologists, and the AUCs of BMSNet, the hematologists, and the pathologists in AML cases were 0.953, 0.965, and 0.997, respectively.

In detecting more than 20% of blasts, the AUCs of the hematologists (0.981) and the pathologists (0.980) were similar and higher than the AUC of BMSNet (0.942). However, the hematologists significantly outperformed BMSNet in detecting more than 20% of blasts. The AUCs of the hematologists (0.981) and the pathologists (0.980) were similar and were higher than the AUC of BMSNet (0.942). The stratified analysis also identified the same trend. However, the differences among BMSNet, the hematologists, and the pathologists were relatively small. Overall, the accuracy of BMSNet was similar to that in real-world clinical practice.

### Human-Machine Competition

**Figure 7:**
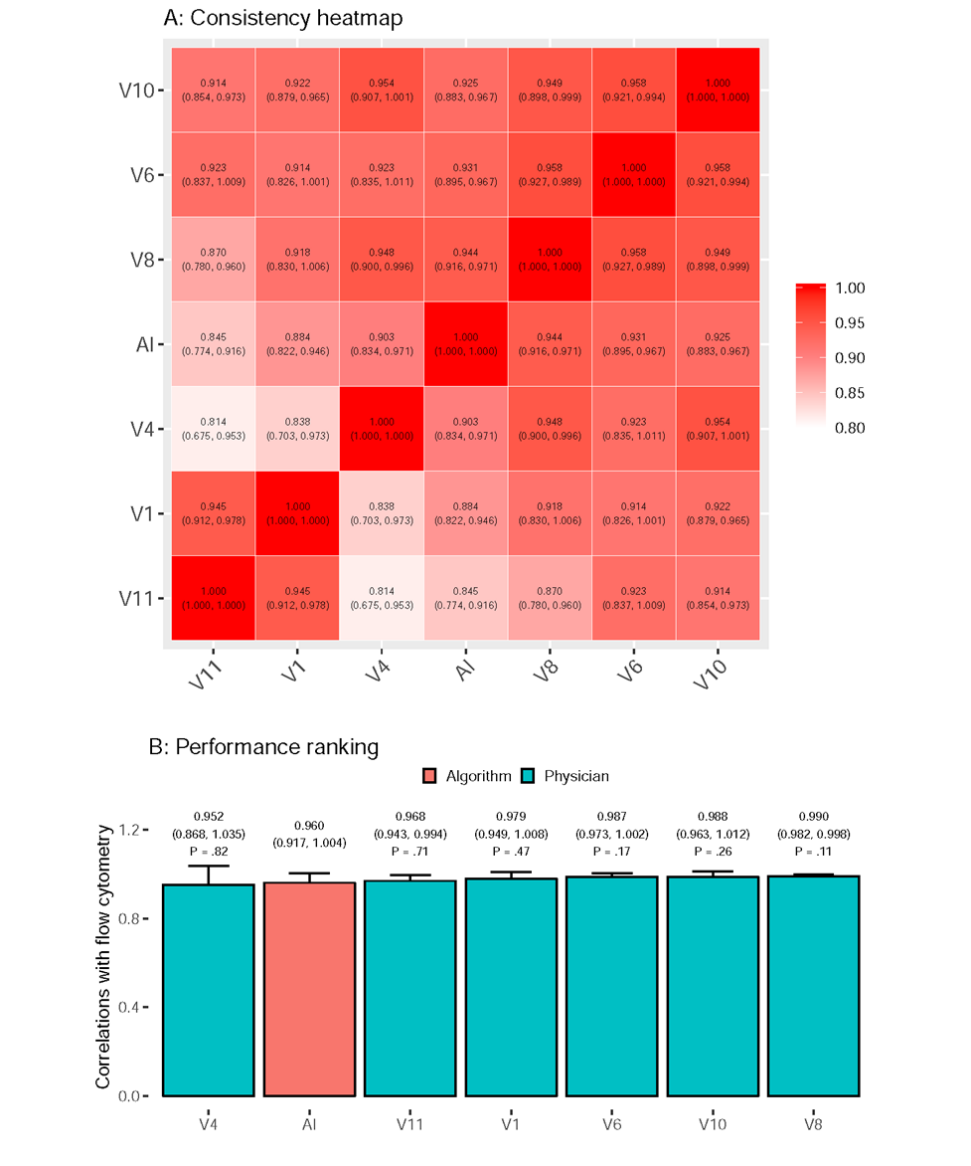
Consistency analysis of the hematologists and BMSNet and their performance rankings in the competition cohort (n=10). (A) Consistency heatmap that is colored according to the values. The values in each cell are the average and the 95% CI of the correlation coefficients. (B) Performance rankings that are based on flow cytometry. The values above the bars are the average values and the 95% CIs of the correlation coefficients and the *P* value for the comparisons between the hematologists and artificial intelligence. The abbreviation V(X) denotes a visiting staff member with (X) years of practice experience.

The results of the human-machine competition are presented in Figure 7. Six visiting staff members were included in this competition. The first analysis was conducted to identify the correlations between the cell proportions that are obtained by BMSNet and humans. The upper part of Figure 7 shows a consistency heatmap, according to which BMSNet is highly consistent with the visiting staff who were teaching it (V10, V8, and V6). Although the correlations within BMSNet and other visiting staff were relatively low, they exceeded 0.845 (V11). This is higher than the lowest correlations among the visiting staff (0.814 in V4 and V11). The second analysis compared the results that were obtained by FCM, as shown in Figure 7. The mean correlation between BMSNet and FCM was 0.960, and the mean correlations between the visiting staff and FCM ranged from 0.952 to 0.990. There was no significant difference between the performances of BMSNet and the visiting staff. This result demonstrated that BMSNet reached the performance level of the visiting staff.

## Discussion

### Principal Findings

BMSNet showed good performance in the interpretation of seven types of cells in bone marrow smears that may be used in the treatment strategy design for acute leukemia. The training plan of BMSNet is initially set to identify the details of more than 20 classifications of each different cell type. However, the trivial maturation differences are difficult to identify, even for well-trained hematological specialists, as reported by a previous investigator [19]. For example, it is typical to identify the same cell as a promyelocyte at first sight but as a myelocyte at the next recognition. Therefore, we merge the cell categories into seven groups plus an *unable to identify* group based on the FCM grouping system for training the AI model. Simplifying the grouping system increased the recognition rate and facilitated comparison with our gold standard, namely, FCM. However, the detailed maturation recognition in the myeloid and erythroid series was abandoned. Dysmorphic features of hematopoietic cells cannot be recognized correctly by the current BMSNet model. This could explain why the performance in terms of the ROC curve is poor for MDS cases. We suggested that the results be reviewed by well-trained hematologists before AI interpretation translates them into clinical data.

On the basis of our ROC curve test, the percentages of blasts correlated well between FCM and the pathologists. As the pathologists also used immunohistochemistry stains for subgroup identification, better performance was realized compared with the hematologists and the BMSNet model. Overall, our BMSNet model performed well, except for MDS with more than 20% blasts. The morphology of blasts in acute leukemia is more uniform and easy to identify, whereas the blasts in MDS are relatively polymorphic, deformed, and difficult to recognize, which may be the cause of the higher misidentification rate of AI compared with the well-trained hematologists and pathologists, who use special immunohistochemistry staining. In addition, several limitations have been identified. The image quality depends on several clinical factors, which include the quality of the bone marrow aspiration, the clinical disease condition, the smear preparation, and the image acquisition. This may cause BMSNet to inaccurately recognize all cells; therefore, the average precision was only 67.4% in cell recognition. However, BMSNet attempts to detect as many objects as possible, including even fragmented cells. Fortunately, these unclear cells were often classified as *unable to identify* and may not affect the performance in clinical practice. This might explain why BMSNet showed a poorer performance in the development cohort than in the validation cohort and the competition cohort.

Acute leukemia is defined as more than 20% blasts in the bone marrow. Therefore, the recognition of blasts is highly important. Furthermore, CD markers facilitate the diagnosis and classification of blasts in identifying the subtypes of leukemia. In FCM, blasts with expressions of CD13, CD33, CD117, and myeloperoxidase are defined as myeloblasts, and those with expressions of CD10, CD19, and terminal deoxytransferase are defined as B lymphoblasts [20]. In the beginning, we planned to identify three kinds of blasts: myeloblasts, lymphoblasts, and monoblasts. However, the variations in the patients’ cell sizes, granularities, and textures hindered recognition, even by a well-trained hematologist. We can increase the recognition accuracy by grouping the blast subtypes. Using BMSNet, we can precisely and quickly detect the percentage of blasts and estimate the leukemia severity. We can quickly evaluate the treatment efficacy of leukemia through BMSNet; however, it is difficult to detect the level of MRD. A larger scale dataset may be needed for the further development of a model for MRD detection.

### Strengths

The performance of our BMSNet model was similar to that of the hematologists. With the AI revolution that was initiated by AlexNet’s victory in 2012 [21], deep learning models have been shown to realize human-level performance and to be effective when large annotated datasets are available [10,22-24]. In several famous cases in the medical field, expert-level performance was also realized, such as in the detection of lymph node metastases [25] and in diabetic retinopathy classification [26]. Our approach realized the same performance in the morphological assessment of bone marrow smears in the validation cohort and in the competition cohort. Several years are needed to train an experienced hematologist, and the performance of BMSNet was at least as high as the performances of the hematologists with more than 1 year of training. A well-trained AI model can help hospitals that lack hematologists and can save a substantial amount of time for experienced hematologists.

### Limitations

Several limitations of this study have been identified. First, the studied photos were captured by experienced technicians, who needed to adjust the focal length and brightness. An optimal process is to use an automatic slide scanner to avoid the operator effect. However, the 1000× photos were necessary for the careful identification of morphological, cytological, and inclusion details [2]. The current best automatic slide scanner can only provide 600× photos. Moreover, we regarded the operation of the microscope as a general technology. This will not affect the application of BMSNet, and other researchers can repeat our work. Second, we compared BMSNet’s performance with those of only six visiting staff members. Although BMSNet and the visiting staff members have realized near-perfect performances compared with the FCM results, comparisons should be made with additional experts to further evaluate the performance of BMSNet.

### Conclusions

In conclusion, we established a deep learning model, namely, BMSNet, for assisting hematologists in reading bone marrow smears. The collaboration between hematologists and AI can save a substantial amount of time and can ensure the consistency of the interpretation results. Moreover, this approach may also facilitate the training of inexperienced students. Future research can expand the database for the detection of additional classes of each cell.

## Abbreviations

AA: aplastic anemia
AI: artificial intelligence
ALL: acute lymphoblastic leukemia
AML: acute myeloid leukemia
AUC: area under the curve
FACS: fluorescence-activated cell sorting
FCM: flow cytometry
IoU: intersection over union
MDS: myelodysplastic syndrome
MM: multiple myeloma
MPD: myeloproliferative disease
MRD: minimal residual disease
ReLU: rectified linear unit
ROC: receiver operating characteristic
WBCs: white blood cells
